# Chest CT target sign in a couple with COVID-19 pneumonia

**DOI:** 10.1590/0100-3984.2020.0089

**Published:** 2020

**Authors:** C. Isabela Silva Müller, Nestor L. Müller

**Affiliations:** 1 Section of Thoracic Imaging - Clínica Delfin, Salvador, BA, Brazil.; 2 Department of Radiology - Vancouver General Hospital, and University of British Columbia, Vancouver, BC, Canada.

**Keywords:** Coronavirus infection, Coronavirus, Computed tomography, Infecção por coronavírus, Coronavírus, Tomografia computadorizada

## Abstract

We describe a target sign on chest CT characterized by a combination of peripheral ring-like opacity and a central nodular ground-glass opacity surrounding a vessel in a couple with COVID-19 pneumonia confirmed by real-time reverse transcriptase fluorescence polymerase chain reaction sputum analysis.

## Abstract (pt)

Descrevemos o sinal do alvo na TC de tórax representando combinação de opacidade com aspecto de anel periférico e componente central nodular em vidro fosco circundando estrutura vascular em um casal com COVID-19 confirmada por reação em cadeia da polimerase com transcrição reversa.

Computed tomography (CT) of the chest is commonly used in the clinical management and assessment of complications of pneumonia caused by the novel coronavirus-severe acute respiratory syndrome coronavirus 2 (SARS-CoV-2)-, as well as to exclude alternative diagnoses^(1)^. Although chest CT plays an important role, it should not be utilized as a screening tool and it can not be used by itself to confirm or exclude the diagnosis of this entity known as COVID-19^(2,3)^, first described in the city of Wuhan, in the province of Hubei, in China, and declared as a pandemic by the World Health Organization in March 2020^(4)^.

The diagnosis of COVID-19 is confirmed by real-time reverse-transcriptase polymerase chain reaction (RT-PCR) and partial or complete sequencing of the viral genome from nasopharyngeal aspirates, nasal and oral swabs, sputum or tracheal or bronchial lavage^(5)^. The vast majority of patients with SARS-CoV-2 is asymptomatic, but the spectrum of clinical presentation is broad including development of severe pneumonia and involvement of various organs.

Chest CT has a high sensitivity (93% to 97%) when the RT-PCR is used as the gold standard for diagnosis, but low specificity (25% to 53%), because of the overlap of the CT findings with those of other pulmonary infections, including influenza H1N1, as well as pulmonary inflammatory processes related to adverse drug reactions, complications of connective tissue disease, vasculitis, etc.^(5-7)^.

The CT findings in symptomatic and asymptomatic patients with COVID-19 and positive RT-PCR have been well described, and are influenced by the stage of the disease, the most common being areas of ground-glass opacity, crazy paving pattern, and consolidation, commonly with a rounded contour and peripheral distribution, but not uncommonly multifocal or diffuse, as well as findings with a pattern suggestive of organizing pneumonia including reverse halo sign and perilobular opacities^(1,5-8)^. Other common manifestations include thickening/enlargement of peripheral pulmonary vessels, commonly within areas of ground-glass opacity^(8-10)^. Less common pulmonary manifestations of COVID-19 include irregular nodules, nodules with CT halo sign, and, in the later stages, bronchial dilatation/distortion and reticulation^(1,5-9)^.

Recently we observed on chest CT a pattern that we called the target sign in a couple with confirmed diagnosis of COVID-19. A 37-year-old woman and her 38-year-old husband underwent chest CT on the same day. The symptoms included fever, that had subsided after a few days, progressive dry cough and, more recently, mild dyspnea. The symptoms had started 12 days previously in the husband and 2 days later in his wife in whom they were more intense. Both had positive RT-PCR for COVID-19 infection. Volumetric noncontrast chest CT of the wife showed moderately extensive parenchymal involvement consisting of predominately peripheral ground-glass opacities, small areas of consolidation and poorly defined nodular opacities. In some peripheral regions of the lung ring-like opacities contained at their center a rounded ground-glass opacity surrounding a vascular structure, a combination of findings that resemble a target (Figure 1). The finding could be seen in cross-sectional, sagittal and coronal planes. The chest CT of her husband showed a similar pattern of abnormalities but only mild parenchymal involvement with a less well-defined target sign in which a ring-like opacity surrounds a subtle area of ground-glass attenuation and in its center a poorly defined nodular ground-glass opacity surrounding a vascular structure (Figure 2).

**Figure 1:**
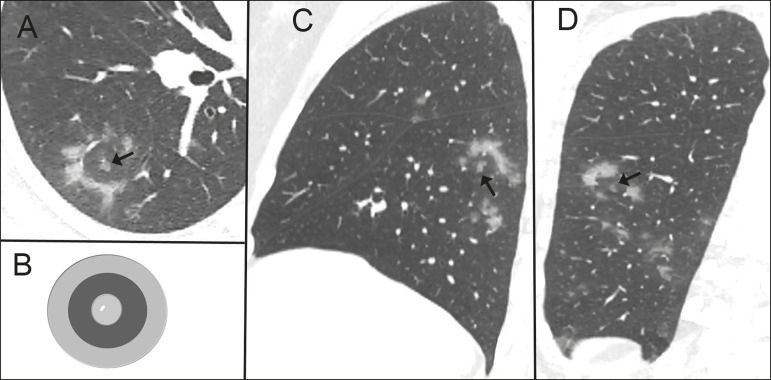
Nonenhanced cross-sectional CT image of the right lung (**A**) in a 37-year-old woman with COVID-19 pneumonia and minimal dyspnea shows an irregular ring-like opacity containing in its center a small nodular ground-glass opacity surrounding a very small vessel (arrow). The combination of central nodular opacity and the peripheral ring of increased opacity resemble a shooting target (**B**) thus resulting in a CT target sign. The target sign can also be seen in the sagittal (**C**, arrow) and coronal (**D**, arrow) reformations. Also noted are small patchy ground-glass opacities.

**Figure 2:**
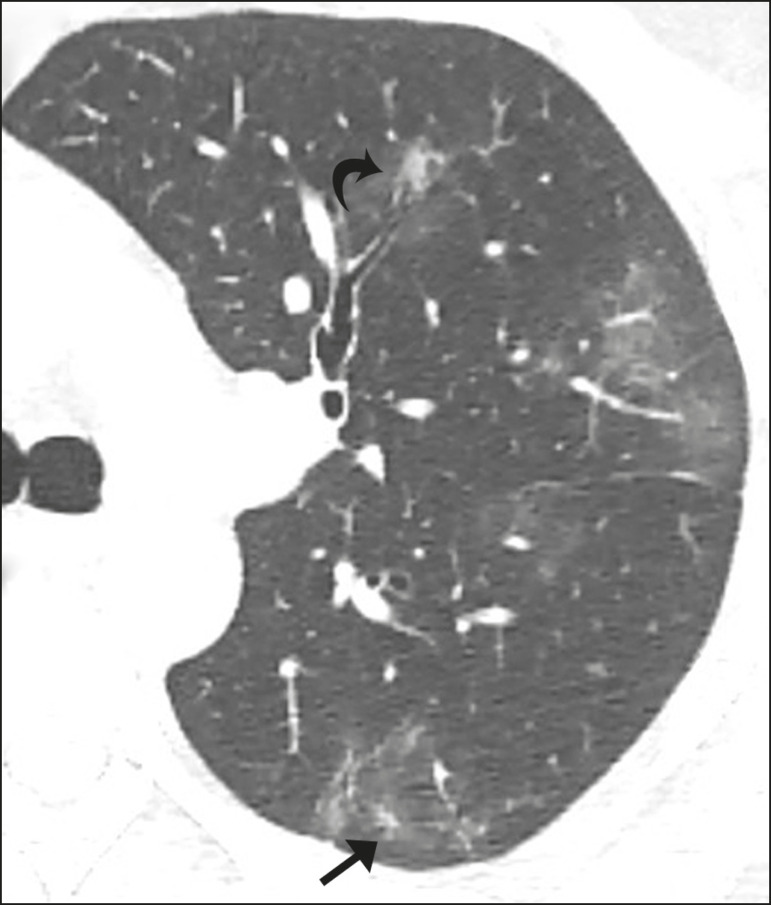
Cross-sectional nonenhanced chest CT image of the left lung in a 38-year-old man (husband of patient showed in Figure 1) with COVID-19 pneumonia shows poorly defined ring-like opacity surrounding a subtle area of ground-glass attenuation and in its center a poorly defined nodular ground-glass opacity surrounding a vascular structure (arrow), resulting in a target sign. Also noted are patchy ground-glass opacities and small poorly defined nodule with halo of ground glass opacity in the left upper lobe (curved arrow).

The CT target sign that we describe here is distinct from the reversed halo sign and the perilobular opacity because of the presence of a nodular opacity in the center of the ring-like opacity. Perilobular opacities and the reversed halo sign are a common finding in organizing pneumonia and have been described in a number of patients with COVID-19 pneumonia^(1,7,8)^. The target sign on chest CT is quite distinct from the target sign seen in diseases of the bowel where it typically reflects the presence of submucosal edema, inflammation, or both^(11)^.

The ring-like opacities in our two patients are suggestive of an organizing pneumonia reaction pattern. The central nodular opacity may reflect the presence of perivascular inflammation and when very dense may represent focal enlargement of the pulmonary artery. Endothelial cell infection and lymphocytic endotheliitis has been demonstrated histologically in post mortem specimens of patients with COVID-19^(12)^. Areas of vascular enlargement or thickening have been described within areas of lung involvement in patients with COVID-19 pneumonia^(8-10)^, but enlargement of small subpleural vessels has also been identified in areas without superimposed opacities in patients with COVID-19, suggesting the presence of diffuse vascular disease^(10)^. In one study that compared COVID-19 to non-COVID-19 pneumonia, vascular thickening was significantly associated with COVID-19 (59% vs. 22%; *p* < 0.001)^(9)^. Similarly, the presence of the target sign on chest CT may be helpful in suggesting the diagnosis of COVID 19 pneumonia in the proper clinical setting. However, confirmation of the potential usefulness of this sign will require further evaluation in a large group of patients comparing the findings of COVID-19 with other causes of infection, particularly viral, and other conditions associated with an organizing pneumonia reaction pattern.
